# Consistency of supplied food and dentition status of the elderly in residential care homes

**DOI:** 10.1186/s12903-019-0770-0

**Published:** 2019-05-02

**Authors:** Yoshiaki Nomura, Ayako Okada, Erika Kakuta, Ryoko Otsuka, Kaoru Sogabe, Kiyoshige Yamane, Taku Yamamoto, Yuko Shigeta, Shuji Shigemoto, Takumi Ogawa, Nobuhiro Hanada

**Affiliations:** 1 Department of Translational Research, Tsurumi University School of Dental Medicine, 2-1-3 Tsurumi, Tsurumi-ku, Yokohama, 230-8501 Japan; 2 Department of Operative Dentistry, Tsurumi University School of Dental Medicine, 2-1-3 Tsurumi, Tsurumi-ku, Yokohama, 230-8501 Japan; 3 Department of Oral Microbiology, Tsurumi University School of Dental Medicine, 2-1-3 Tsurumi, Tsurumi-ku, Yokohama, 230-8501 Japan; 4 Yamane Dental Clinic KUNSHIKAI Medical Corporation, 7-9, Tennojiya, Yao-shi, Osaka, 581-0025 Japan; 5 Supercourt Co, Ltd, 1-7-7, Nishihommachi, Nishi-ku Osaka-shi, Osaka, 550-0005 Japan; 6 Department of Fixed Prosthodontics, Tsurumi University School of Dental Medicine, 2-1-3 Tsurumi, Tsurumi-ku, Yokohama, 230-8501 Japan

**Keywords:** Supplied food consistency, Care level, Tooth contact, Removable denture

## Abstract

**Background:**

The association between oral health and malnutrition has been investigated in detail. The nutrition of elderly subjects in residential care homes is determined by caregivers, dietitians or nutritionists and managed by changing the consistency of their supplied food. However, few reports have described the relationship between oral condition and supplied food consistency. The objective of this study was to determine dentition status and care levels that correlate with supplied food consistency among elderly residents of care facilities. In addition, we estimated the care level at which ordinary food consistency can be supplied by caregivers who cannot diagnose dental status.

**Method:**

Several factors, including dentition, wearing removable dentures, meals categorized as ordinary or processed (sliced, mashed, or liquefied), and care levels according to the Japanese standardized care-needs certification system were investigated in 276 elderly residents (male, *n* = 56; female, *n* = 220; mean age, 87.68 ± 5.94 years) of 12 fee-based care facilities.

**Results:**

The results of this study showed that care levels were significantly correlated with the consistency of the food supplied to the residents. When supplied food consistency was categorized as ordinary or processed, the number of remaining teeth and the number of tooth contact pairs, either natural or artificial, were statistically significant. From logistic regression analysis, it was determined that the numbers of tooth contact pairs were statistically significant among residents requiring high levels of care.

**Conclusion:**

The number of tooth contact pairs, either natural or artificial, was one of the contributing factors for deciding supplied food consistency among elderly residents of care facilities. Elderly residents requiring less than care level 3 should have ordinary meals.

**Electronic supplementary material:**

The online version of this article (10.1186/s12903-019-0770-0) contains supplementary material, which is available to authorized users.

## Background

Mastication is an important physical function for nutrition intake, metabolism, and health-related quality of life, especially among the elderly. Masticatory efficiency is affected by the presence of teeth, the number of functional teeth, prostheses, and functional decline. All of these exert influence over food choices [1–3]. Dietary limitations due to chewing difficulties as a result of tooth loss or ill-fitting dentures can result in impaired nutritional status [4–11]. Three cross-sectional and longitudinal studies have identified a significant association between oral health status and food intake [3, 9, 12].

Diet plays an important role in disease prevention [13–15]. Dietary limitations and inadequate dentition can be the etiology of common systemic diseases, such as bowel cancer and coronary heart disease, especially among the elderly [7, 16].

Several studies have confirmed an association between inadequate dentition and malnutrition [17–22]. However, dietitians or nutritionists who manage diets by changing the consistency of supplied food are in the position to administer nutrition to elderly residents of nursing homes and residential care facilities. Caregivers observe the leftovers after the residents’ meals and decide what types of food consistency are needed to minimize the effort of mastication, chewing and swallowing. Ordinary, sliced, mashed, or liquefied foods are served according to caregivers’ decision.

Ideally, dietitians or caregivers should diagnose the quality of dentures and poor mastication. Even in a super-aging Japanese society in which one of every five residents is over the age of 65 years and with dependable coverage for dental visits provided by the national insurance system, dentists, in general, do not concern themselves with nutrition and the consistency of a patient’s diet. The knowledge of dentistry that caregivers, nutritionists or dietitians have is not still enough. Even though a nutritionist or dietician can determine a food supply that is nutritionally sufficient, inappropriate food consistency can lead to an elderly person leaving a dish unfinished. This will result in a lack of sufficient nutritional conditions, and finally, lead to malnutrition. One of the obstacles in this situation results from absence of a tool for communication between the dentist and the caregivers, dietitians, or nutritionists. There is no guideline regarding the consistency of food supplied to elderly residents in nursing homes and residential care facilities. In addition, there is no fact-finding survey on the current situation regarding dental status and food consistency, even in a super-aging Japanese society.

Mastication is an important function that is directly tied to health-related quality of life. In fact, items concerning mastication have been included in representative questionnaires to investigate oral health-related quality of life [23–26]. In addition, mastication is associated with brain activity [27–30] and stress management [31]. Mastication is essential for good health-related quality of life and to prevent systemic diseases. Therefore, the consistency of supplied food should not simply be down-regulated according to complaints or requests from elderly residents of care facilities.

Several studies have demonstrated the association between oral health status and malnutrition. However, little information available regarding the consistency of supplied food. As far as we can ascertain, only one study had shown a significant correlation between denture use and supplied food consistency [32]. The study focused on denture use, not supplied food consistency; therefore, more detailed information about the consistency of the supplied food was not available. Consequently, there is no information to support clear decision criteria for changing the consistency of supplied food based on dentition status. In this study, we set up food consistency as a major outcome. The present cross-sectional survey investigated the relations between dentition status and supplied food consistency among elderly residents of care facilities.

## Materials and methods

### Study design and setting

A cross-sectional study was carried out to investigate the consistency of food supplied for the elderly residents of 12 care facilities. These facilities were located around the Osaka area in Japan. The correlation between supplied food consistency and the number of remaining teeth, number of tooth contact pairs in the premolar and molar regions, and care levels were investigated.

### Participants

We enrolled 276 elderly residents (male, *n* = 56; female, *n* = 220; mean age, 87.68 ± 5.94 years) of 12 fee-based care facilities who provided written, informed consent before the start of data collection to participate in the present study. This study was noninvasive, and there were no interventions; therefore, we did not set up any exclusion criteria except to exclude those potential subjects who could not understand the aims of this study. Residents who were unable to understand the study aims or to provide written, informed consent because of dementia or other conditions were excluded (*n* = 10).

### Sample size estimation

For the sample size calculation concerning supplied food consistency, as far as we are aware, information from only one previous study was available [32]. The authors investigated 205 subjects in a nursing home; among them, 181 subjects were classified as needing dentures, which was defined as subjects with fewer than 20 remaining teeth. By a cross-tabulation of denture use and supplied food consistency (ordinary or processed), the sample size was calculated. To determine the statistical significance, it was necessary to have 39 subjects for each group. From the medical record form for visits in the regular care system, described in the following section, information about the number of remaining teeth was already available before the start of this study. To enroll more than 39 subjects with fewer than 20 remaining teeth, we decided to include 12 facilities.

The sample size estimated above was appropriate to investigate the relation between supplied food consistency and denture use; however, this outcome was not consistent with major outcome of this study. Therefore, we additionally calculated a post hoc sample size for Table 4. We calculated the sample size necessary to obtain statistically significant odds ratios for the dependent variables: the number of natural teeth/natural teeth contact, natural teeth/denture contact, and dentures/denture contact when included in logistic regression analysis for the subjects with care levels 3, 4, and 5 (*n* = 145). After calculation of the standard deviations of the number of natural teeth/natural teeth contact pairs, natural teeth/denture contact pairs, and dentures/denture contact pairs, their co-relation with ordinary or processed food was calculated by Spearman’s ρ. The calculations were carried out by R software with the Power/Sample Size Calculation for Mediation Analysis package, using following commands.

$$ {\displaystyle \begin{array}{c}\begin{array}{l}\mathrm{ssMediation}.\mathrm{VSMc}.\mathrm{logistic}\Big(\mathrm{power}=0.80,\mathrm{b}2=\log\ (1.43),\mathrm{sigma}.\mathrm{m}=1.87,\mathrm{p}=0.5,\mathrm{corr}.\mathrm{xm}=\hbox{-} 0.1,\\ {}\mathrm{alpha}=0.05,\mathrm{verbose}=\mathrm{TRUE}\Big)\end{array}\\ {}\begin{array}{l}\mathrm{ssMediation}.\mathrm{VSMc}.\mathrm{logistic}\Big(\mathrm{power}=0.80,\mathrm{b}2=\log\ (1.42),\mathrm{sigma}.\mathrm{m}=2.14,\mathrm{p}=0.5,\mathrm{corr}.\mathrm{xm}=0.004,\\ {}\mathrm{alpha}=0.05,\mathrm{verbose}=\mathrm{TRUE}\Big)\end{array}\\ {}\begin{array}{l}\mathrm{ssMediation}.\mathrm{VSMc}.\mathrm{logistic}\Big(\mathrm{power}=0.80,\mathrm{b}2=\log\ (1.26),\mathrm{sigma}.\mathrm{m}=3.54,\mathrm{p}=0.5,\mathrm{corr}.\mathrm{xm}=0.037,\\ {}\mathrm{alpha}=0.05,\mathrm{verbose}=\mathrm{TRUE}\Big)\end{array}\end{array}} $$

The results were 70.9, 55.8, and 47.0 samples for natural teeth/natural teeth contact, natural teeth/denture contact, and dentures/denture contact, respectively. Therefore, we decided the sample size was enough for the purpose of this study.

### Medical and dental treatment and check-ups

The 12 facilities in our study were administered by one corporation. Medical and dental care was controlled by one medical corporation. Before moving into the care facilities, all subjects were obligated to undergo medical and dental checkups. After moving into the care facilities, patients had access to a medical doctor and dentist who visited the facilities once or twice a week. Medical and dental treatment was carried out by the visiting medical doctor or dentist if necessary. Health and oral conditions of the elderly at the care residences were checked at least once a month. The quality of the medical and dental care including denture quality, was kept almost constant level with through the regular visiting care system. Regular professional cleaning of the teeth and oral mucosal cleaning (i.e., tongue cleaning) by a dental hygienist were provided for all the residents. Therefore, there were no patients with an extremely inadequate dental status resulting from a lack of dental treatment or inadequate dentures. However, some subjects refused to wear dentures. For these subjects, dental caries and periodontal disease were treated if necessary. In addition, regular professional cleaning of the teeth and tongue were provided.

### Oral examination

Three dentists conducted oral examinations to determine the presence or absence of teeth, their locations, use of removable dentures, and number of tooth contact pairs in the premolar and molar regions, including dentures, at least once a month. The data used in this study were the results of oral examinations conducted October 1, 2016.

### Meals

The care facilities offered residents buffet meals based on a standard menu and nutritional quality. Caregivers selected the texture of each meal, categorized as ordinary, sliced, mashed or liquefied, according to the caregivers’ decision, which was based on the observation of leftovers or on requests from individual residents, not on advice from dental staff.

### Care levels

The national insurance system covers the care of all elderly individuals in Japan. Care-needs certification and determination of care levels (Certification of Needed Long-Term Care) are standardized. Several reports have described this system in detail [33–36].

Computer software initially calculates standardized scores for elderly applicants based on 79 items that include seven dimensions of physical and mental status and the type of assistance required. The amount of time required to implement nine categories of care is estimated; then, care-needs levels are assigned based on total estimated values.

The Nursing Care Needs Certification Board and a group of physicians, nurses, and other experts in health and social services determine whether the initial assessment of the applicant was appropriate, considering the opinion of the person’s primary care physician. Finally, the board determines care levels. Care levels are assigned to categories including support requirements 1 and 2, and are levels 1 to 5. At this stage, dementia or other diseases are diagnosed by the primary care physician. In addition, the care levels are updated by every six months. A summary of the care levels defined by the Japanese Certification of Needed Long-Term Care are as follows:

- Support requirement 1: Eating and excretion can be done by oneself, but assistance is necessary for some kind of daily living activities such as cleaning.
- Support requirement 2: The ability to participate in daily living activities declines from the state of support described in requirement 1, and some kind of support or partial care is required.
- Care level 1: Eating and excretion can be done by oneself, but care is necessary for daily activities such as standing up, etc.
- Care level 2: Care is partially necessary for eating and excretion, and care is necessary for most activities of daily living including standing up and walking.
- Care level 3: Excretion, activities of daily living and standing up cannot be done by oneself. In some cases, walking by oneself cannot be done.
- Care level 4: Most of the activities of daily living, including excretion and standing up, cannot be done by oneself. In some cases, behavior cannot be controlled, and a decline in cognition is observed.
- Care level 5: In addition to the details of are level 4, eating cannot be done by oneself.

### Variables

The variables analyzed in this study were the consistency of supplied food, care levels, number of remaining teeth, and the number of tooth contact pairs in the premolar and molar regions and their types. In some analyses, consistency of supplied food and care levels were dichotomized. Supplied food consistency was dichotomized as either an ordinary meal or a processed meal.

### Statistical analysis

*P* values for cross tabulations were calculated using Fisher’s exact test, and statistically significant cells in cross tabulations were determined by log-linear analysis. Significant differences in the numbers of remaining teeth and/or number of tooth contact pairs according to supplied food consistency were determined using the Mann-Whitney U or Kruskal-Wallis tests. Ordinary and processed meals were included as dependent variables in logistic regression analyses to eliminate the effect of care levels. Optimal cutoff values for care levels for ordinary and processed meals were determined by ROC analysis.

Data were statistically analyzed using IBM SPSS Statistics (Version 22.0; IBM SPSS, Tokyo, Japan).

## Ethics

The Ethics Committee of the Tsurumi University School of Dental Medicine approved this study (approval number, 1329), which proceeded in accordance with the Declaration of Helsinki.

## Results

Among the 276 subjects participating in this study, 199 (72.1%) wore removable dentures and 75 (27.2%) were edentulous. The numbers of remaining teeth were 8 (0–16) for median and 25^th^ to 75^th^ percentile and 9.23 ± 8.66 for mean and standard deviation. Supplied food consistency and dental status are summarized in Table 1. The median and 25^th^–75^th^ percentiles and mean and standard deviation of dental status against supplied food consistency are presented. The number of remaining teeth and number of tooth contact pairs in the premolar and molar regions were higher for the subjects who were supplied ordinary meals than for those who ate processed meals. All values were all statistically significant by the Mann-Whitney U tests.

**Table 1:** Cross tabulation of number of remaining teeth and tooth contact pairs in premolar and molar regions against supplied food consistency

Supplied food consistency							
	Ordinary meal (*n* = 145)	Processed meal (*n* = 131)	P*	Processed meal			P**
				Sliced (*n* = 107)	Mashed (*n* = 18)	Liquefied (*n* = 6)	
Number of remaining teeth	9 (0–18)	7 (0–13)	0.048	7 (0–14)	5 (0–13)	3 (0–20)	0.389
	10.0 ± 8.9	8.2 ± 0.7		8.4 ± 8.4	7.2 ± 7.7	8.2 ± 10.6	
Presence of antagonistic teeth in premolar and molar regions							
Natural teeth/natural teeth	0 (0–2)	0 (0–0)	0.019	0 (0–0)	0 (0–0)	0 (0–4)	0.164
	1.2 ± 1.9	0.8 ± 0.2		0.8 ± 1.8	0.7 ± 1.8	1.3 ± 2.2	
Natural teeth/denture	0 (0–3)	0 (0–2)	0.041	0 (0–2)	0 (0–2)	0 (0–0)	0.484
	1.5 ± 2.2	1.1 ± 0.2		1.2 ± 1.8	1.1 ± 1.8	0 ± 0	
Dentures/denture	3 (0–8)	2 (0–8)	0.047	2 (0–8)	4 (0–8)	0 (0–2)	0.484
	3.5 ± 3.5	3.3 ± 0.3		3.3 ± 3.6	3.7 ± 3.6	± 3.2	

Data are shown as medians (25^th^–75^th^ percentiles) and as the means ± SD. Number of remaining teeth decreased among residents who consumed processed meals, except for liquefied meals. When comparing ordinary and processed meals, the difference in the number of remaining teeth was statistically significant. Numbers of tooth contact pairs in the premolar and molar regions were also statistically significant. However, for food consistency divided into ordinary, sliced, mashed and liquefied, differences were not significant *Mann-Whitney U test, ** Kruskal Wallis test

**Fig. 1:**
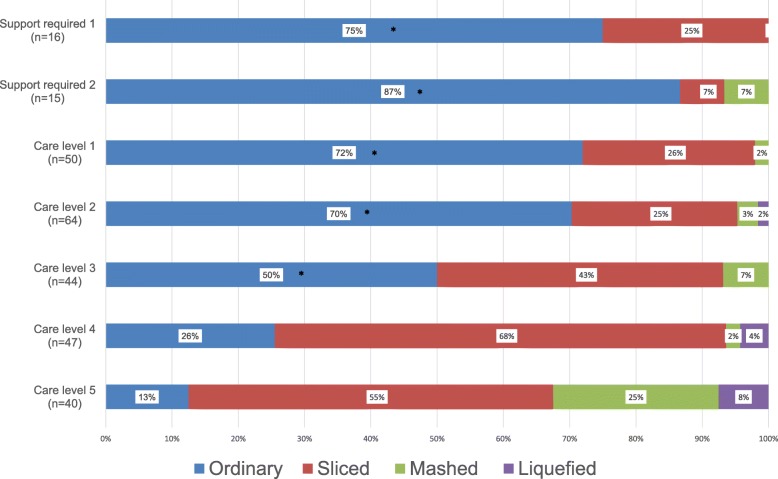
Distribution of prescribed food consistency against care levels for the 276 elderly residents of the selected care facilities. The distribution was statistically significant by Fisher’s exact test (*P* < 0.001); * indicated the statistically significant cells by log-linear model (*P* < 0.05). Frequency of ordinary diet in support levels 1 and 2 and care levels 1, 2, and 3 are statistically significant (log-linear analysis). Most elderly residents requiring support levels 1(12/16, 75.0%) and 2(13/12, 86.6%) consumed ordinary meals. In contrast, most elderly residents requiring care levels 4 (35/47, 74.5%) and 5 (35/40, 87.5%) consumed processed meals

The care levels were evaluated by daily living activities; therefore, care levels may be an important confounder of supplied food consistency. We analyzed the co-relation between supplied food consistency and care levels. Figure 1 shows the distribution of supplied food consistency against care levels. Care levels were statistically significantly associated with supplied food consistency (*P* < 0.001, by Fisher’s exact test). The proportion of subjects who consumed processed meals increased concomitantly with increasing care levels. Most participants requiring support levels 1 and 2 consumed ordinary meals, whereas only 5 (12.5%) of the 40 who required care level 5 consumed ordinary meals. Mashed and liquefied food were rare for subjects determined to be at less than care level 4. By log linear analysis, for the subjects at care levels less than 3, the proportion of supplied ordinary meals was statistically significant.

From the results of Table 1 and Fig. 1, both dental status and care level had statistically significant co-relations with supplied food consistency. To evaluate the effect of dental status, multiple logistic regression analyses were necessary. In addition, as shown in Table 1, the results of log-linear analysis had shown that supplied ordinary meals were not statistically significant for the subjects with care levels 4 and 5. This result indicated that the effect of dental status may differ in the subjects in different care levels. Another aim of this analysis was to evaluate the effect of dental status between different care levels.

Odds ratios for consuming processed food by the number of remaining teeth and the number of tooth contact pairs in the premolar and molar regions adjusted by care levels were calculated. As shown in Fig. 1, dose response relations were observed between supplied food consistency and care levels; we regarded the care levels as continuous variables. Table 2 shows the results of crude and multivariate adjusted odds ratios obtained by logistic regression analysis. The crude odds ratio of care level was statistically significant by simple logistic regression analysis. The crude odds ratio of the number of remaining teeth was not statistically significant. Odds ratios of all types of number of tooth contact pairs were statistically significant. The adjusted odds ratio of for the number of remaining teeth was not statistically significant (Model 1). When the number of tooth contact pairs was adjusted by care level, the number of natural teeth/natural teeth type contacts was statistically significant (*P* = 0.017), whereas natural teeth/dentures and dentures/dentures were not significant (*P* = 0.063 and *P* = 0.068, respectively).

**Table 2:** Logistic regression analysis of supplied food consistency by care levels, number of remaining teeth, and number of tooth contact pairs in premolar and molar regions

			Model 1		Model 2	
	Crude odds ratio (95% CI)	P	Adjusted odds ratio (95% CI)	P	Adjusted odds ratio (95% CI)	P
Care level	1.909 (1.589–2.294)	< 0.001	1.908 (1.586–2.294)	< 0.001	1.865 (1.546–2.250)	< 0.001
Number of remaining teeth	1.026 (0.998–1.055)	0.070	1.025 (0.993–1.057)	0.127		
Number of tooth contact pairs in premolar and molar regions						
Natural teeth/natural teeth	1.323 (1.110–1.577)	0.002			1.263 (1.043–1.531)	0.017
Natural teeth/dentures	1.259 (1.089–1.455)	0.002			1.167 (0.991–1.374)	0.063
Dentures/dentures	1.139 (1.039–1.249)	0.006			1.101 (0.993–1.221)	0.068

As shown in Fig. 1, are levels were strong confounders for supplied food consistency when considering that mastication is a major function of food consistency and that teeth are a major functional device for mastication. Supplied food consistencies were dichotomized as ordinary or processed food. By simple logistic regression analysis, tooth contact pairs in the premolar and molar regions were more important than the number of remaining teeth. When adjusted by care level, the number of remaining teeth was not statistically significant (Model 1). For the tooth contact pairs in the premolar and moral regions, only natural teeth/natural teeth contact was statistically significant (Model 2). The *p*-values of natural teeth/dentures and dentures/dentures contact were not statistically significant. However, *p*-values of these contact style were close to 0.05

The optimal cutoff point of care levels associated with ordinary and processed meals was assessed by ROC analysis. The optimal cut off point existed between care levels 2 and 3. The participants were assigned to groups according to care level, and the logistic regression analyses were repeated (Table 3). The odds ratios of the number of tooth contact pairs in the premolar and molar regions for consuming processed meals were all statistically significant for subjects requiring care levels 3, 4, and 5, but not for those requiring support levels 1 and 2 and care levels 1 and 2.

**Table 3:** Logistic regression analysis of ordinary and processed meals according to number of tooth contact pairs in the premolar and molar regions for subgroups by care levels

	Odds ratio (95% CI)	P
Support required 1 and 2 and Care levels 1 and 2 (*n* = 133)		
Natural teeth/natural teeth	1.16 (0.85–1.77)	0.341
Natural teeth/dentures	1.06 (0.84–1.33)	0.581
Dentures/dentures	1.03 (0.87–1.20)	0.722
Care levels 3, 4, and 5 (*n* = 145)		
Natural teeth/natural teeth	1.43 (1.13–2.25)	0.004
Natural teeth/dentures	1.42 (1.11–1.95)	0.004
Dentures/dentures	1.26 (1.10–1.55)	0.003

Effect of the number of tooth contact pairs on supplied food consistency by subgroup analysis by care levels. Care levels were dichotomized by ROC analysis. Food consistency was affected by the number of tooth contact pairs for the subjects with intensive care levels. In contrast, food consistency was not affected by the number of tooth contact pairs for subjects who needed partial care for daily living, including eating

For the subgroup of care levels 3, 4 and 5, post hoc sample size calculations were carried out. The sample size to obtain statistically significant odds ratios were 71, 55 and 50 samples for the number of natural teeth/natural teeth, natural teeth/dentures, and dentures/denture contact, respectively

To determine the characteristics of participants who did not consume ordinary meals, a table was constructed to assess relationships among numbers of remaining teeth and the presence or absence of dentures against supplied food consistency (Table 4). The results of log-linear analyses showed a statistically biased distribution among participants with 1–5, 6–10, and 11–15 remaining teeth and those without dentures. The frequency of consuming ordinary meals was lower in these participants.

**Table 4:** Supplied food consistency against number of remaining teeth and presence or absence of dentures

		Supplied food consistency	
Remaining teeth (n)	Dentures	Ordinary meal	Processed meal
0	–	0^*^	1
	+	38	36
1–5	–	1^*^	10
	+	19	15
6–10	–	1^*^	6
	+	18	17
11–15	–	3^*^	7
	+	18	12
16–20	–	10	6
	+	14	6
21+	–	21	11
	+	2	4
Total	–	36	41
	+	109	90

*Statistically significant difference, P < 0.05, (log-linear model). Subjects with fewer than 15 remaining teeth and not wearing dentures were significantly unlikely to consume ordinary meals

## Discussion

Here, we investigated the dental status of elderly residents of care facilities and the consistency of their supplied food. According to the national statistics of Japan, there were 86,000 nursing home for elderly and 4,400,000 elderly persons were resided in nursing homes at 2017 all over Japan [37]. Food consistency is one of the important factors for maintaining mastication in the elderly; however, little information is available regarding food consistency, and no fact-finding survey exists. The Japanese Society of Dysphagia Rehabilitation presents a classification system of food for patients with dysphagia. For the swallowing training, suitable food consistency were classified as by their property, configuration and viscosity. This classification is not yet international and is aimed at patients with dysphagia. In a sense, this guideline is focused on treatment and rehabilitation training for patients with dysphagia. There is no adequate guideline regarding the consistency of food supplied to elderly individuals without dysphagia. Therefore, there are no criteria for dietitians or caregivers, and there is no tool for communication with a dentist. Even though nutritionists or dieticians supply nutritionally adequate controlled food, inappropriate food consistency may lead to an elderly person leaving a dish unfinished. This can cause insufficient nutritional conditions, leading, finally, to malnutrition. In contrast, as described in the Introduction, mastication may have several effects on the health of the elderly [23–31]. Chewing ability and dentition status are influenced on the mortality and frailty of elderly persons [38–41]. Individuals with impaired dentition demonstrated a significantly greater degree of decline in the intake of multiple nutrients (protein, sodium, potassium, calcium, vitamin A, vitamin E and dietary fiber) and food groups (vegetable and meat) than those without impaired dentition [38]. Reduced chewing ability of soft foods increased the risk of mortality [40]. Masticatory function evaluated by mixing ability and subjective chewing ability of were significantly related to progression to frailty or pre-frailty [41]. To maintain the masticatory function, caregivers should not change supplied food consistency based only on complaints or requests from elderly residents. However, there is no standardized guideline to determine the consistency of the food supplied.

In this study, care levels were the most important determinant for supplied food consistency, and care levels were decided based on ADL and assistance, including help with dietary intake. Dentition status was also important because the number of tooth contact pairs in the premolar and molar regions were statistically significant for supplied food consistency after adjustment by care levels. Especially, for the subjects with care levels 3, 4 and 5, the number of tooth contact pairs were important for deciding supplied food consistency, even when the antagonistic teeth were dentures.

Many studies have suggested that the degree of edentulousness affects the quality of nutrient intake. Dentate elderly had a significantly higher nutritional quality of the diet than edentulous or denture wearers [42]. .The maximal bite force is 5–6-fold lower among edentulous denture wearers [43] and the chewing efficiency of denture wearers is 54% that of dentate subjects [44]. However, ingenious cooking and dental treatments can prevent nutritional deficiencies among individuals with reduced masticatory efficiency. Odds ratios for the number of tooth contact pairs for supplied food consistency were all statistically significant for participants in the present study who required care levels 3, 4, and 5. These results indicated that the number of tooth contact pairs in the premolar and molar regions is important for the subjects with a care level greater than 3, even when the antagonistic teeth are dentures. Our results agree with those of other studies; removable partial dentures effectively improve dietary intake, even among individuals who have lost only a few teeth. They consumed more vegetables, n-3 fatty acids, calcium, vitamin A, and dietary fiber than non wearers [45]. However, the odds ratios of the number of tooth contact pairs was not statistically significant for participants who required support levels 1 and 2 and care levels 1 and 2, indicating that such persons should consume ordinary meals. In fact, > 70% of participants who required support levels 1 and 2 and care levels 1 and 2 did consume ordinary meals. Caregivers should not change supplied food consistency based only on complaints or requests from elderly residents, even when tooth contact is insufficient or dentures are ill-fitting.

Log-linear analysis showed that the association between denture use and supplied food consistency differed significantly among participants who consumed ordinary meals, those who did not wear dentures, and those who had fewer than 15 remaining teeth. The ability of dependent elderly persons to use dentures is associated with their degree of physical and mental capacity: ability to dress/undress and ability to rinse the mouth [32], cognitive impairment [46], attitudes to dentists, dentures, and dentistry [47], behavior evaluated by geriatric behaviour-rating scale [48], with and without dementia [49], and mental status [50]. Subjects requiring care levels 3 and above might find the use of dentures difficult. However, the physical condition of edentulous patients who do not wear dentures significantly deteriorates, and the six-year mortality rates of edentulous elderly residents without dentures are considerably higher than those of the residents with 20 or more teeth in Japanese nursing homes [51]. Dentures thus seem necessary for the elderly to maintain their health and quality of life.

We were unable to assess swallowing function in the present study. Even the repetitive saliva or modified water swallowing tests could pose a risk of aspiration pneumonia for some residents [52]. Swallowing function might be another important factor for deciding the appropriate consistency of supplied food [53]. Additionally, this study was cross-sectional. We could not decide causal relations. Additional longitudinal studies are needed to examine interactions among dentition status, swallowing function, and supplied food consistency.

## Conclusion

Tooth contact, including that made by removable dentures, was one of the contributing factors for determining supplied food consistency in elderly residents of care facilities. In addition, caregivers, nutritionists or dieticians should not change the consistency of supplied food only because of a request from elderly residents. Dentist dentists should participate in the discussion when deciding the supplied food consistency along with caregivers, nutritionists or dieticians. In addition, elderly residents with a care level less than 3 should have ordinary meals.

## Additional file

Additional file 1: Raw data. (XLSX 76 kb)

## Abbreviations

Certification of Needed Long-Term Care: Care-needs certification and determination of care levels
